# Protein phosphorylation detection using dual-mode field-effect devices and nanoplasmonic sensors

**DOI:** 10.1038/srep08687

**Published:** 2015-03-03

**Authors:** Nikhil Bhalla, Mirella Di Lorenzo, Giordano Pula, Pedro Estrela

**Affiliations:** 1 Department of Electronic & Electrical Engineering, University of Bath, Bath BA2 7AY, United Kingdom; 2 Department of Chemical Engineering, University of Bath, Bath BA2 7AY, United Kingdom; 3 Department of Pharmacy & Pharmacology, University of Bath, Bath BA2 7AY, United Kingdom

## Abstract

Phosphorylation by kinases is an important post-translational modification of proteins. It is a critical control for the regulation of vital cellular activities, and its dysregulation is implicated in several diseases. A common drug discovery approach involves, therefore, time-consuming screenings of large libraries of candidate compounds to identify novel inhibitors of protein kinases. In this work, we propose a novel method that combines localized surface plasmon resonance (LSPR) and electrolyte insulator semiconductor (EIS)-based proton detection for the rapid identification of novel protein kinase inhibitors. In particular, the selective detection of thiophosphorylated proteins by LSPR is achieved by changing their resonance properties *via* a pre-binding with gold nanoparticles. In parallel, the EIS field-effect structure allows the real-time electrochemical monitoring of the protein phosphorylation by detecting the release of protons associated with the kinases activity. This innovative combination of both field-effect and nanoplasmonic sensing makes the detection of protein phosphorylation more reliable and effective. As a result, the screening of protein kinase inhibitors becomes more rapid, sensitive, robust and cost-effective.

To regulate various cellular activities, proteins undergo post-translational modifications. These modifications cause conformational changes in the structure and activity of proteins *via* chemical addition of specific moieties to target amino acids within proteins (*e.g.* phosphate in the case of phosphorylation, carbohydrates in the case of glycation and glycosylation, etc)^1^. Protein phosphorylation is the addition of phosphate groups to a protein, which is catalysed by kinases. It regulates almost all aspects of cell life, such as increasing or suppressing enzymatic activities; marking a protein for degradation; regulating protein trafficking; modulating protein-protein interactions. Because of the importance of protein phosphorylation in cell regulation, functional perturbation of kinase activities results in a variety of diseases^2^,^3^. In this respect, the discovery of molecules able to modulate protein kinase and especially their inhibitors is of extreme interest for the development of new drugs^4^. To meet this need, researchers routinely use mass spectroscopy^5^, phosphor-specific antibody^6^ and radioisotope labelling^7^. These techniques present several limitations that considerably slow down the development of efficient protein kinase-targeting drugs. Mass spectrometry requires large investments and expertise, besides demanding a lot of attention in the preparation of samples^8^. The use of phosphor-specific antibodies is also expensive and relies on the development of reliable target-specific antibodies^9^. Moreover, the detection methods (*i.e.* ELISA or immunoblotting) are time-consuming. Radioisotope-labelling entails the use of expensive and hazardous reagents and is not easily available for all investigators. In recent times, some groups have described the development of sensitive and selective electrochemical^10^,^11^,^12^,^13^,^14^,^15^ and optical detection methodologies^16^ for investigating kinase activity. These methodologies offer several advantages in terms of reagent requirement, multiplexing and screening throughput, adaptability to different kinase targets, routine cost and time for the analysis. However, the development of technologies that enable efficient and convenient analyses of protein phosphorylation *in vitro* and are suitable for screening of large libraries of candidate compounds has not been achieved yet.

Previous electrochemical methods attempted the detection of protein phosphorylation by measuring one of the following two chemical events: the addition of negative charges to the protein with the transfer of phosphoryl groups^17^,^18^; the release of protons in the reaction buffer upon phosphorylation of protein^19^. There have been also attempts to detect the changes in the charge of the protein after phosphorylation by measuring the alterations on the surface charge of an electrode in contact with the protein, which is recorded in the form of current as a function of time^17^,^18^. We recently reported the analysis of protein phosphorylation by measuring the release of protons using electrolyte insulator semiconductor (EIS) sensors and by measuring direct pH change using commercial micro electrodes^19^.

This study reports for the first time a dual-mode sensor that uses an electrolyte–insulator–semiconductor (EIS) field-effect device coupled with nanoplasmonic effects measured by a localized surface plasmon resonance (LSPR) technique within the same experimental platform. During phosphorylation of proteins, the phosphate group at the γ-position of the adenosine triphosphate (ATP) is transferred to the serine, threonine or tyrosine amino acids of the protein^18^. If 5'-[γ-thio] triphosphate (ATP-S) is utilized in kinase assays, proteins are thio-phosphorylated (*i.e.* conjugated to a phosphate containing a sulfhydryl group replacing a hydroxyl group). Thio-phosphorylated proteins present the remarkable ability to bind to gold nanoparticles (AuNPs), which allows their electrochemical^18^,^20^ and optical detection.

In this study, the detection of protein kinase C alpha (PKC-α) activity is used as a model to demonstrate the applicability of the dual-mode AuNP-based LSPR and EIS sensors. PKC-α has been found implicated in several disease states, including cancer^22^,^23^, heart failure^24^,^25^ and bipolar disorder^26^,^27^. The phosphorylation of myelin basic protein (MBP) by PKC-α is a well-characterised protein phosphorylation reaction for which potent inhibitors are known. In our experimental model, GF 109203X, a known inhibitor of PKC-α, was utilised to confirm the specificity of the technique proposed and to prove that this system can be employed to discover novel protein kinase inhibitors. The thio-phosphorilation of MBP, followed by AuNPs binding, causes a wavelength shift in the resonance, which can be detected by LSPR. Furthermore, the use of silicon nitride as a pH sensitive surface enables the formation of a two-in-one sensing platform by integrating LSPR detection with EIS sensing. This dual detection mechanism for phosphorylation enables real-time monitoring and a double cross-checked complementary validation for the activity of new inhibitor under test, with the use of very low amounts of kinase (down to 10 mU/μl). The technique proposed can also be easily miniaturised to allow further reductions of the volume of reagents required.

## Results and Discussion

**Figure 1:**
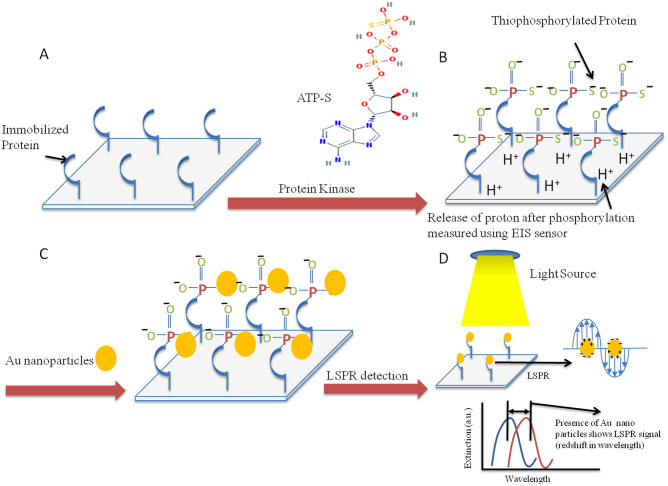
Schematic of working principle. (A) Immobilized protein on silicon nitride surface. (B) Upon thio-phosphorylation there is a release of proton (which allows EIS-dependent monitoring of the reaction) and transfer of γ phosphate from ATP-S. (C) Covalent attachment of AuNPs to the sulphide group on thio-phosphorylated protein. (D) LSPR detection mechanism.

The schematic of the phosphorylation reaction in our experimental conditions is presented in Figure 1. MBP was immobilized on a Si_3_N_4_ (Si-SiO_2_-Si_3_N_4_) wafer using 3-glycidoxypropyltrimethoxysilane (GOPTS) for 1 h at room temperature inside a customised teflon well; subsequently thio-phosphorylation of MBP by the PKC-α kinase was carried out in the presence of ATP-S. Three sets of control reactions were performed: one without PKC lipid activator; a second with the GF109203X inhibitor added before putting the kinase activator; and a third reaction where the protein phosphorylation was carried with ATP instead of ATP-S. Upon phosphorylation of MBP in the presence of ATP-S, the protein becomes thio-phosphorylated. The thio-phosphorylated proteins were then exposed to AuNPs (16 nm diameter average size), wherein the affinity between the thiol groups and Au resulted in the attachment of AuNPs to the thio-phosphorylated substrates. Generally, the covalent attachment of quasi-spherical AuNPs to the thiol groups occurs at a fast rate so after 10 min the substrates were rigorously washed with the reaction buffer to remove electrostatically attached AuNPs. As a consequence, the phosphorylated protein was labelled with AuNPs that enabled its detection through LSPR, alongside pH changes on the EIS substrate.

**Figure 2:**
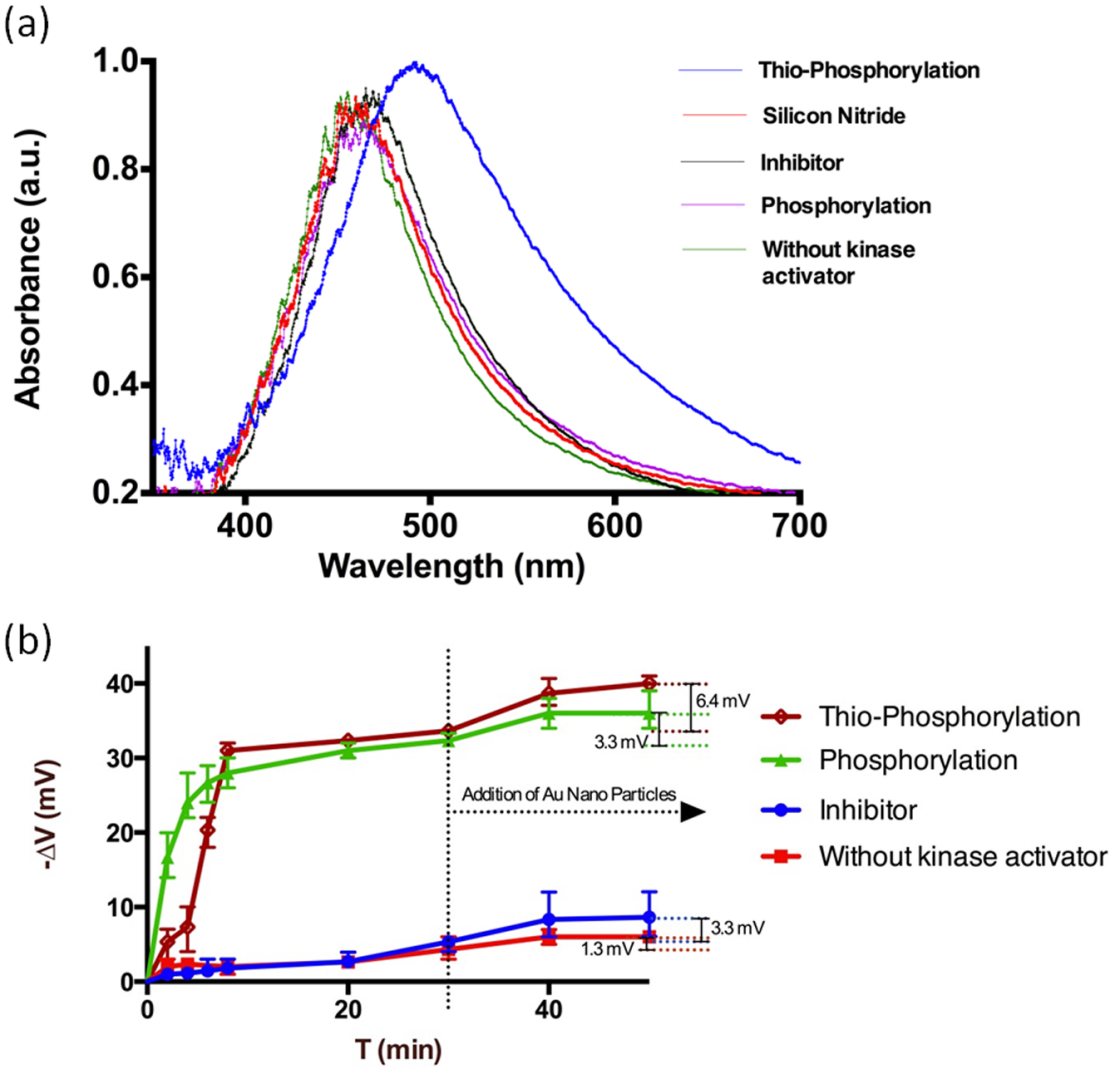
Time courses of LSPR and EIS detection of phosphorylation on Silicon Nitride. (a) LSPR spectra in the absorption mode for samples thio-phosporylated and controls (without kinase activator, ATP phosphorylation, and in the presence of an inhibitor), as well as for the Si_3_N_4_ background. (b) Kinase activity detected by measuring the release of proton associated with protein phosphorylation for the same samples shown in 2a.

As shown in Figure 2a, a shift of 31 nm in the absorption spectrum, compared to the silicon nitride background, is observed in the thio-phosphorylated sample. In the absence of kinase activator, a 10–12 nm shift in wavelength was observed. This indicated some non-specific adsorption of AuNPs to the sample. Nevertheless, this result suggests that the majority of the shift in wavelength of the thio-phosphorylated sample was due to the covalent attachment of AuNPs to the sulphide group present on the γ-phosphate group transferred from ATP-S to the MBP (*i.e.* it is a reliable measurement of PKC-α activity). The reaction where ATP-S was replaced by ATP (*i.e.* a normally phosphorylated sample) showed around 9 nm of average wavelength shift, thus confirming non-specific binding of AuNPs to MBP (*i.e.* independent of thio-phosphorylation). This phenomena was however significantly lower than the true positive signals but did not affect the measurements. When the reaction was performed in the presence of a known inhibitor of kinase, GF109203X, a 7 nm of shift in wavelength was observed. These results support the hypothesis that inhibitors could be screened using the LSPR approach.

As the chemical reaction was performed on Si_3_N_4_, protein thio-phosphorylation could also be followed electrochemically as we previously described^19^. In the present work, we measured the release of proton associated with the protein ATP-S thio-phosphorylation in a similar way to what was reported previously for standard ATP phosphorylation^19^. Thio-phosphorylated samples showed a shift in the Capacitance–Voltage characteristics 30–35 mV very similar to samples in which phosphorylation was obtained in the presence of ATP, and significantly higher than negative controls in the absence of kinase or in the presence of kinase inhibitor (Figure 2b). It was also observed that the rate of transfer of γ-phosphate from ATP-S was slower than normal phosphorylation in the presence of ATP (25 mV at 2 minutes *vs* 5 mV at the same time point). This result is consistent with previous reports^28^,^29^.

**Figure 3:**
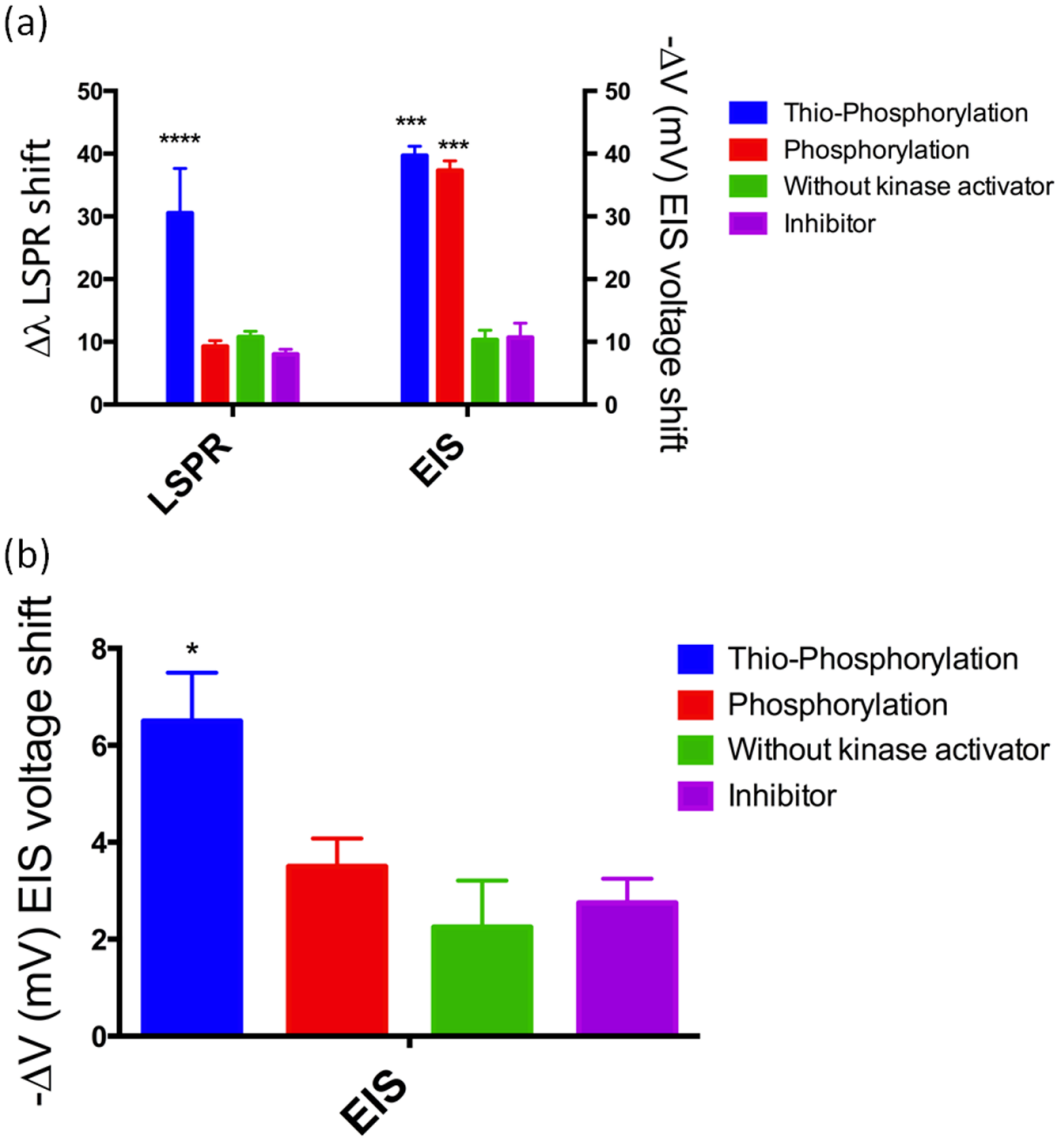
(a) Statistical analysis of LSPR and EIS-based detection of thio-phosphorylation. Thio-phosphorylation data (blue) resulted significantly different from controls without kinase (green) for both LSPR- and EIS-based analyses (* = p < 0.05 n = 3). Phosphorylation results (red) were not statistically different from the controls without kinase for LSPR detection, but statistically significant for EIS-based analyses. Thio-phosphorylation reaction in the presence of PKC inhibitor GF109203X (purple) did not elicit detection levels significantly higher than control in the absence of kinase (green). (b) Statistical analysis of change in EIS signal as a consequence of AuNPs binding (Bonferroni post-test). The thio-phosphorylation was found as significant when compared to the other reactions.

The experiments shown in Figure 2 were repeated for three independent times. The average data are presented and analysed by one-way ANOVA with Bonferroni post-test (Figure 3). The signal obtained by both LSPR and electrochemical detection of proton release resulted significant for thiophosphorylation compared to negative controls (without kinase activator or in the presence of inhibitor, p < 0.05). As expected, phosphorylation did not provide a significant signal in the LSPR experiments, while it did in the EIS-based measurements. The wavelength shift in thio-phosphorylated samples is due to the plasmonic oscillations of electrons in the AuNPs. There is also an increased absorbance observed in the thio-phosphorylated samples which is due to radiative decay of strongly enhanced electric near field of the AuNPs that scatters light on silicon nitride, allowing it to absorb more energy in the far field at the LSPR frequency. As shown in Figure 2b, the addition of AuNPs had some minor effects on the detection of phosphorylation and thio-phosphorylation by EIS. The overall voltage shifts due to AuNP addition for thio-phosphorylation, phosphorylation, mock phosphorylation (*i.e.* no kinase activator) and phosphorylation in the presence of PKC inhibitor are shown in Figure 3b, which highlights a change in the voltage observed for thio-phosphorylation of 6.4 mV. This result can be attributed to the fact that the attachment of AuNPs slightly disrupts the outer Helmholtz plane (OHP) of the double layer capacitance in the Gouy-Chapman-Stern-Graham model of the EIS sensor. As a result, the adsorption of ions from the OHP onto the electrode surface is triggered by constant Coulombic attractions forming the inner Helmholtz plane (IHP). This gives rise to surface complexation^30^, whereby the affinity of attracting counter ions from the buffer becomes higher, allowing the formation of complex compounds on the surface. This, in turn, brings about slight changes in the surface potential in that vicinity.

**Figure 4:**
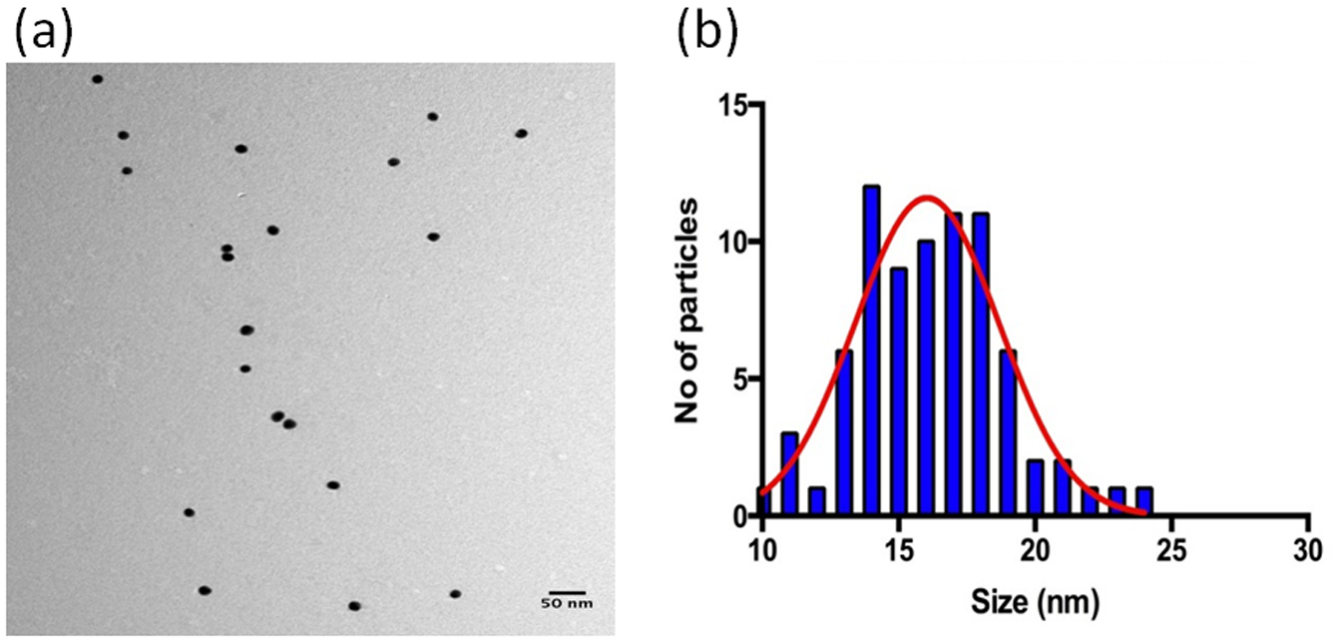
(a) TEM image of thio-phosphorylated sample. (b) Size distribution of AuNPs, obtained by processing the TEM images of the AuNPs with the Image-J software.

Interestingly, the variation observed in the LSPR shifts from sample to sample was larger in the case of thio-phosphorylated samples than to the EIS signal for the same samples. The variation in the LSPR signals can be attributed to: 1) inconsistency in the shape and size of the nanoparticles; 2) non-homogeneity of the nanoparticles; 3) variable density of AuNPs from sample to sample to which LSPR signal is sensitive. TEM analysis of the thio-phosphorylated substrates confirmed the irregularity in the shape and size of the nanoparticles in addition to variable density and heterogeneity among individual AuNPs in form of tetrahedral, cubic, octahedral and hexagonal phases that was apparent from the scans (Figure 4a). The spatial diameter of the AuNPs in two dimensions, analyzed using Image-J, varied from 10 nm to 25 nm in Gaussian distribution curve with average size of 17–18 nm (Figure 4b). Producing AuNPs of consistent shape, size and homogeneity in bulk solution, is practically impossible due to small variations in the equilibrium of the Gibbs free energy during the nucleation and growth of nanoparticles in solution. However, the variable density in the distribution of AuNPs, for instance random Au-Au couples that were observed in some areas, was ascribed to the formation of aggregates due to the increased affinity between the peptides and AuNPs in the presence of ATP-S^31^ and the non-uniform distribution of the immobilized proteins that hinders the attachment of gold to all the thio-phosphorylated sites. Additionally, pairs of AuNPs are observed at some sites, which are likely to form a ‘plasmonic ruler' configuration. According to the equation of the plasmonic ruler for the Au particle pair trapped in a protein medium^32^, the corresponding LSPR shift should vary between 20 and 40 nm for the Au-Au distance given the size of particles observed under TEM. This is consistent with our observations: when thio-phosphorylation takes place, an LSPR shift in the order of 30 nm is observed. It should be noted that, even with heterogeneity of the AuNPs observed, non-specific interactions of AuNPs to the samples only yield shifts in the order of 10 nm.

Since LSPR shits depend on the size of AuNPs^33^, the effect of the AuNP size in our system was studied using AuNPs of 5, 10 and 20 nm diameter (nominal sizes as provided by the supplier). In order to isolate the effect of AuNP size from variations in phosphorylation amounts, Si_3_N_4_ samples were silanized with 3-mercaptopropyl triethoxysilane (MPTES), which provides a free thiol group on the surface for direct AuNP binding. A full monolayer of AuNPs (or AuNPs aggregates) is expected with this surface chemistry. Upon immobilization of AuNPs, LSPR shifts of 19.3 ± 1.6 nm, 23.3 ± 2.2 nm and 31.3 ± 2.5 nm were observed for AuNPs of diameter 5 nm, 10 nm and 20 nm, respectively. These results indicate that the AuNP heterogeneity observed in Figure 4 can indeed be responsible for the large error bars in the LSPR signals observed upon thio-phosphorylation, but that even with AuNPs of 10 nm size (the minimum size observed in the TEM images), shifts for a surface saturated with AuNPs are well above those observed for the control experiments. Therefore the heterogeneity of the AuNPs does not play a significant role in the system.

The physical dimensions of the AuNPs are much larger than those of MBP. It is therefore possible that a similar number of AuNPs is attached for different amounts of thio-phosphorylation. In order to test for this possibility, the density of proteins immobilized on the surface can be controlled and checked for thio-phosphorylation with LSPR. To control the distribution of proteins on the surface, self-assembled monolayers (SAMs) of APTES/AHS (3-amino propyl triethoxy silane and allyl hydroxl silane) with 0.01%, 0.1%, 1%, 10%, and 100% of concentration of APTES were tested. This leads to coatings with different density and protein distribution that can be used to test the effect of protein density distribution on thio-phosphorylation detection using our EIS/LSPR technology. In order to check the effectiveness of this new method of controlling protein immobilization by using different solution ratios of APTES/AHS, horse radish peroxidase (HRP) was used as a test protein. Initial experiments were performed by immobilization of HRP with varied concentrations of APTES and testing the levels of HRP using a TMB (3,3',5,5'-Tetramethylbenzidine) assay. (Figure S3 in supplementary information). Higher concentration of APTES in APTES/AHS led to a higher amount of HRP immobilized on the surface of silicon nitride. However, the linearity was low (regression coefficient <0.97), especially at higher concentrations of APTES possibly due to the linear mismatch in the orientation of how SAMs are formed on the surface and the way proteins attach to the functionalized group on the SAM. Using a similar immobilization approach, an analysis was performed on how LSPR- and EIS-based phosphorylation detection were affected by APTES concentration in the immobilization of MBP (Figure S4 in supplementary information). Our assumption that density of MBP can be controlled in a similar way as that of HRP was validated when the EIS signal was found different at varied concentrations of APTES with optimal level only at 100% APTES; the LSPR signal at 1% 10% and 100% concentrations of APTES was found to be near-maximal. This might suggest that the amount at 1% concentrations of APTES the amount of immobilised protein is sufficient for maximal AuNP binding and saturation of the LSPR signal.

**Figure 5:**
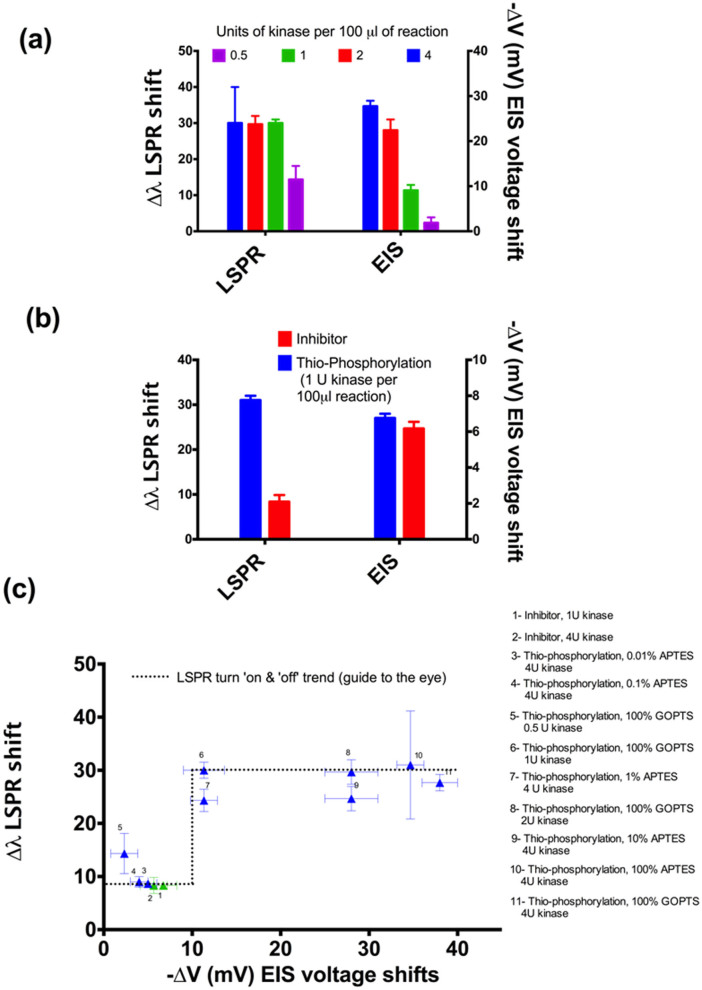
(a). EIS and LSPR response at varied concentrations of APTES in APTES/AHS solution used for the immobilization of MBP. (b) Comparison of assay efficiency at different kinase concentration on EIS and LSPR in the presence or absence of 0.1 μM PKC inhibitor. (c) Variation of LSPR shifts with respect to the corresponding EIS shits – all samples and controls are presented.

In addition, the effect of varying concentrations of kinase on the assay efficiency was studied using both LSPR and EIS (see Figure 5a). The response of LSPR was found to be marginally more sensitive than the EIS technique, which allowed the detection of a significant signal at low concentrations of kinase. At 10 mU/μl the LSPR response is in fact maximal, whereas the EIS response is only around 30% of the maximum observed at 100 mU/μl of kinase. The EIS response is also linear at low concentrations of kinase whereas the LSPR detection is typically dichotomic (*i.e.* on-off response, possibly suggesting saturation of binding sites for AuNPs at low thio-phosphorylation levels). To validate the sensitivity of LSPR for the identification of kinase inhibitors at this concentration of kinase, thio-phosphorylation experiments were conducted in the presence of PKC inhibitor (0.1 μM) – in this case both the LSPR and EIS signals are at their minimum (Figure 5b). This response shows complementary sensing capabilities of EIS and LSPR. At lower concentrations of kinase or when low amount of phosphorylation happens, EIS will fail to distinguish signals from controls. On the other hand, LSPR confirms the phosphorylation reaction with a digital response, *i.e.* irrespective of the amount of phosphorylation LSPR distinguishes controls from the reaction in study provided that at least 10 mU/μl kinase is utilized in the reaction. Figure 5c shows the results for all the experiments including controls: while EIS signals vary according to the amount of thio-phosphorylation, LSPR gives an on-off response, confirming that low EIS signals are indeed due to phosphorylation. It is worth noting that the only point where a partial ‘on' LSPR signal was observed is for 5 mU/μl of kinase (point #5 in Figure 5c), where no EIS signal is observed but LSPR shows that a small amount of phosphorylation does occur. The Si_3_N_4_ samples were also analysed using Fourier transform infrared spectroscopy (FTIR) in reflection mode, which confirmed the presence of strong sulphide bonds in the thio-phosphorylated samples. Strong peaks of sulphide were observed at 1090 nm where the sample was phosphorylated in presence of ATP-S (please see supplementary information). However, this peak was absent in the control reactions. The other peaks seen on the silicon nitride corresponded to the Si groups in GOPTS and the MBP.

## Conclusions

This study describes a new method for detecting phosphorylation of proteins. Owing to the unique optical properties of AuNPs, a simple LSPR-based technique coupled to a field-effect device was developed for the evaluation of kinase activity and inhibition. LSPR gives a ‘on-off' response upon thio-phosphorylation; even at low concentrations of phosphorylation where EIS gives a small positive response, LSPR can be used to validate it as a true signal. Our approach demonstrates that multiple sensing techniques can be employed on the same sensing platform to achieve high levels of sensitivity and accuracy in the measurements. We also propose a novel method for controlling immobilisation of proteins via silanization by changing solution ratios of APTES/AHS. The combined detection method that we propose can easily be extended to multiplexed approaches (*i.e.* microplates) for high throughput analyses of protein kinase activity. As a result, the screening of protein kinase inhibitors becomes more rapid, sensitive, robust and cost-effective. This is paramount for drug discovery in pharmaceutical industries and academia.

## Methods

### Reagents

All chemicals were of analytical grade and were used as received, unless otherwise specified. All aqueous solutions were made with double de-ionised water, 18.2 MΩ cm, with a Pyrogard filter (Millipore, USA). Tris base, MgCl_2_, NaCl, acetone, NH_4_OH, HCl, H_2_O_2_, 3-glycidoxypropyltrimethoxysilane (GOPTS), PKC-α kinase inhibitor GF 109203X, adenosine tri-phosphate (ATP), adenosine 5'-[γ-thio] triphosphate (ATP-S), 3-mercaptopropyl triethoxysilane (MPTES), 3,3′,5,5′-tetramentylbenzidine (TMB), allyl hydroxyl silane (AHS) and horseradish peroxidase (HRP) were purchased from Sigma-Aldrich. Dephosphorylated myelin basic protein (MBP), purified from bovine brain using FPLC liquid chromatography, was purchased from Millipore. Protein Kinase C alpha (PKC-α) human recombinant kinase produced in Sf9, was procured from ProSec-Tray TechnoGene Ltd. PKC lipid activator cocktail was obtained from Millipore. Polyclonal phospho-(Ser) PKC substrate antibody was purchased from Cell Signaling Technology. 20 nm research grade gold nanoparticles (AuNPs) were bought in colloidal form from Diagnostic Consulting Network.

### Si_3_N_4_ sample preparation

The Si_3_N_4_ samples were prepared in the same way as described in a previous report^21^. Briefly, 100 nm of Si_3_N_4_ was deposited by plasma-enhanced chemical vapour deposition (PECVD) onto 4-inch n-type Si wafers with 50 nm of SiO_2_. The Si-SiO_2_-Si_3_N_4_ wafer was cleaned using a standard RCA wafer cleaning protocol. After cleaning, 100 nm aluminium was physically deposited on the back of the Si wafer to serve as an ohmic back-contact using an Edwards e-beam evaporator. The Si_3_N_4_ surface was then cleaned using acetone vapour at 110°C. For the electrolyte–insulator–semiconductor measurements, the wafer was sandwiched between a teflon well with an o-ring and a conductive plate (copper), so that the aluminium coated side of the wafer sits on the lower conductive plate. This formed a planar Si_3_N_4_ well with 19.64 mm^2^ interrogation area for the reaction defined by the size of the o-ring (5 mm diameter).

### Phosphorylation on Si_3_N_4_

The protein was immobilized on the Si_3_N_4_ as described in a previous report^21^. Phosphorylation of MBP was carried out in a buffer with low ionic strength (0.2 mM Tris base, pH 7.4, 6 mM NaCl and 0.4 mM MgCl_2_). The whole reaction volume was fixed to 100 μl for all replicates and controls. 1 μM ATP-S and 4 units of PKC-α (40 mU/μl)) were subsequently added. To initiate the phosphorylation reaction, PKC lipid activator (1:20 of reaction volume) containing 0.5 mg/ml phosphatidylserine and 0.05 mg/ml diacyglycerol in 20 mM MOPS (pH 7.2), 25 mM β-glycerol phosphate, 1 mM sodium orthovanadate, 1 mM dithiothretiol and 1 mM CaCl_2_, was added. Three sets of control reactions were performed, one without PKC lipid activator, second with 0.1 μM PKC kinase inhibitor (GF 109203X), that were added before adding the kinase activator and the third reaction where protein phosphorylation was carried with ATP instead of ATP-S. 30 minutes after the start of the reaction, AuNPs were added and later the substrates were rinsed rigorously with reaction buffer to remove unbound gold nanoparticles.

### LSPR detection and microscopy

The instruments for LSPR namely, reflection probe (R400-7UV-VIS), halogen light source (LS-1-LL) and the spectroscope (USB4000-UV-VIS-ES) were purchased from Ocean Optics. Before taking any signal from the scope, the system was calibrated for dark and light spectrum modes. The LSPR signal was then recorded in absorption mode by observing the wavelength dependence of the light absorbed through by nanoparticles *via* the SpectraSuite software (cross-platform spectroscopy operating software from Ocean Optics). Transmission electron microscopy (TEM) was done at 200,000× with JEOL JEM1200EXII transmission electron microscope with high contrast pole pieces. For TEM, 300-mesh nickel grids, coated with a fine layer of carbon, were used as substrates for the protein fractions in the microscope. MBP was immobilized in the same way as described in the above sections. The Fourier Transform Infrared Spectroscopy (FTIR) was done directly over the surface of Si_3_N_4_ using a Perkin Elmer Frontier FTIR instrument in reflection mode and a high resolution Mercury Cadmium Telluride (MCT) detector.

### Capacitance–Voltage characterisation

PKC-α activity was measured by analysing Capacitance–Voltage (*C*–*V*) characteristics of the Si_3_N_4_/SiO_2_/Si EIS structure. *C*–*V* measurements were performed using a CompactStat digital potentiostat (Ivium Technologies, The Netherlands). A conventional three-electrode electrochemical setup was employed with an Ag/AgCl reference electrode immersed in the electrolyte *via* a salt bridge used to apply the gate voltage and a Pt counter electrode. During the measurements, the gate voltage (*V*_g_, applied between the reference electrode and the Al back-contact) was varied between −2 and +4 V, superimposed with a small ac signal of 10 mV at 1 kHz. The first measurement for the reaction was taken after adding ATP and kinase, *i.e.* before the start of phosphorylation process. After adding the kinase activator, the activity of the reaction was studied by recording the *C*–*V* characteristics every 2 minutes for 10 minutes. Finally the measurements were taken at 20, 40 and 50 minutes after the start of phosphorylation. In control reactions, *C*–*V* measurements were taken at the same time intervals. Each experiment was repeated at least three times and the reported data correspond to the average values.

## Author Contributions

N.B. conducted the experiments, prepared all figures and wrote the main part of the manuscript text. M.D.L., G.P. and P.E. supervised different aspects of the work and contributed to the writing of the manuscript text. All authors reviewed the manuscript.

## Supplementary Material

Supplementary Information Supplementary Information
