# Supplementary material for: Protein phosphorylation detection using dual-mode field-effect devices and nanoplasmonic sensors
Source: Sci Rep. 2015 Mar 3;5:8687. doi: 10.1038/srep08687 (PMC4346972; doi:10.1038/srep08687)
Supplement: Supplementary Information [file srep08687-s1.pdf]

SUPPLEMENTARY INFORMATION

Protein phosphorylation detection using dual-mode field-effect devices and nanoplasmonic sensors

Nikhil Bhalla, Mirella Di Lorenzo, Giordano Pula, Pedro Estrela

Kinase Assay

Figure S1 shows a schematic of the thio-phosphorylation assay, including protein immobilization and LSPR measurement steps.

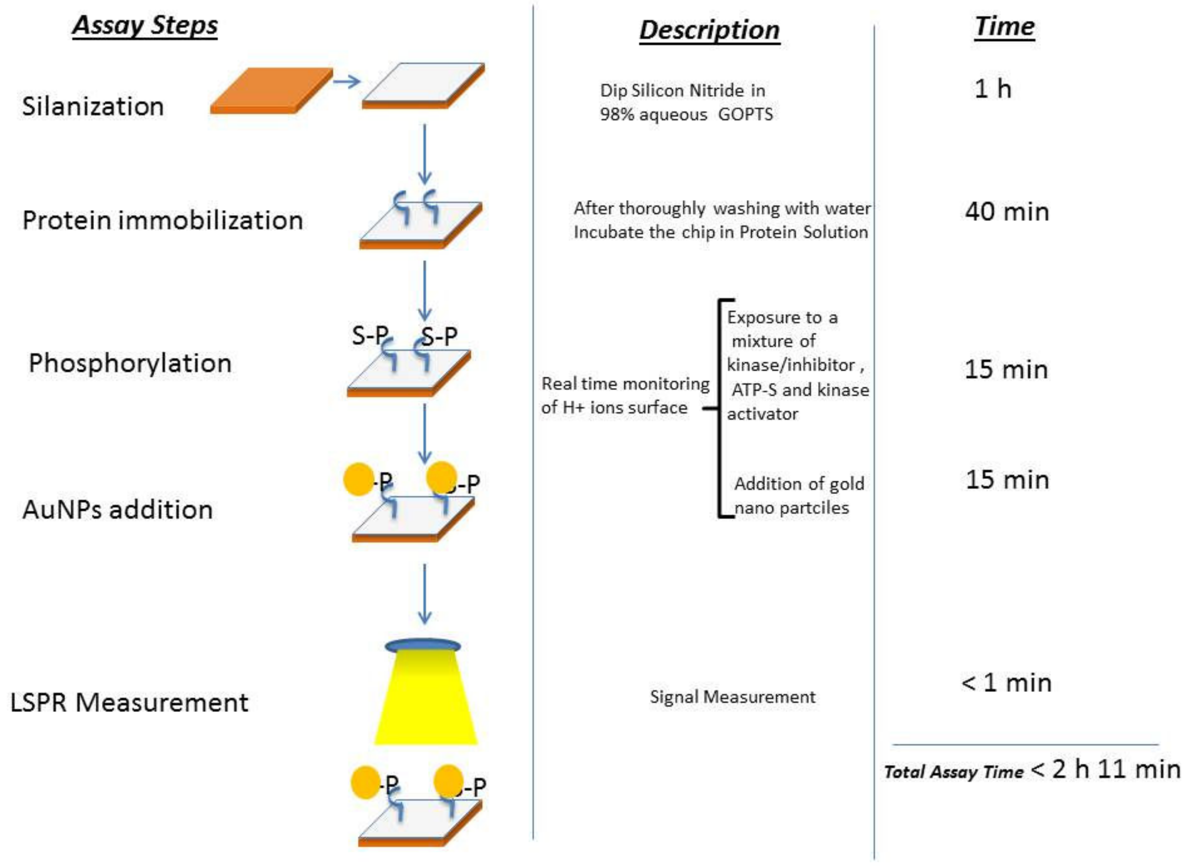

Figure S1 - Kinase Assay Schematic

FTIR analysis of Si_3N_4 samples after phospholyration:

The Fourier Transform Infrared Spectroscopy (FTIR) was done directly over the surface of Si_3N_4 using a Perkin Elmer Frontier FTIR instrument in reflection mode and a high resolution Mercury Cadmium Telluride (MCT) detector. Figure S2 shows the FTIR spectra for samples

with MBP immobilized after different phosphorylation conditions including controls. The list of peaks observed is presented in Table S1.

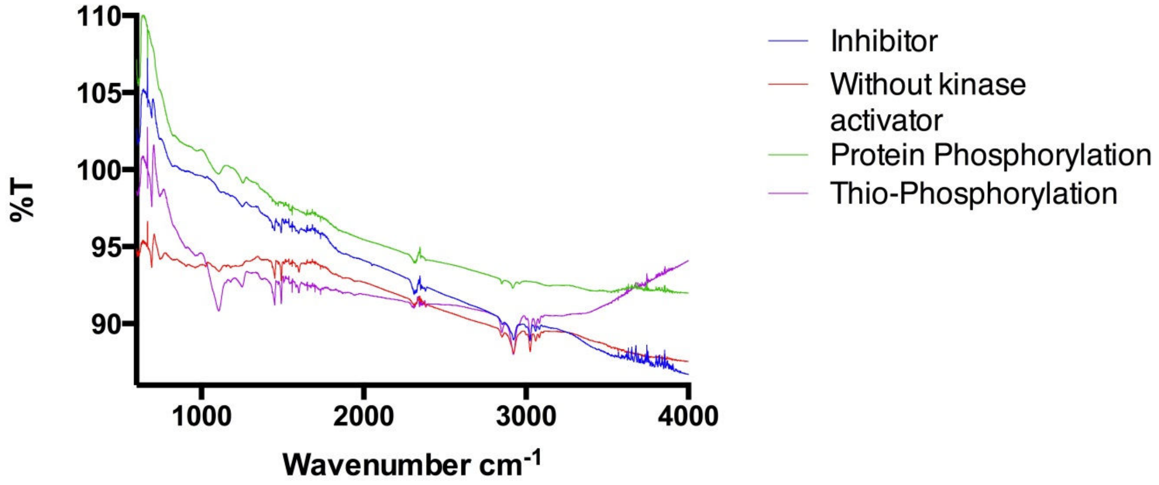

Figure S2 - FTIR analysis on silicon nitride in reflection mode.

Table S1 - Relevant FTIR peaks observed

Peaks (cm^{-1})	Thio-phosphorylation	Phosphorylation	Without Kinase Activator	With Inhibitor
1090 , presence of P, S bonds	Present	Small peak is seen indicating absence of S bonds	Absent	Absent
1225 , phosphoamide bonds	Present	Present	Absent	A small peak relatively insignificant in comparison to phosphorytated sample
1443,1452 Alkanes, Sulphide bond	Very strong peak indicating presence of Suphide and alkanes from GOPTS and MBP	Present	Present	Present
Between 1500-1700 Carboxylic acids and its derivatives	Present	Present	Present	Present
2275, 2300 , Carboxylic acids, overlap of C-H, C=H C=C bonds and Si-H bonds	Present	Present	Present	Present
2850, 2915 Aldehydes,	Present	Present	Present	Present
3000-3050 boarding of O-H bonds stretched by AuNPs	Presents	Absent	Present	Present

Control of protein density via silanization:

To control the distribution of proteins on the surface, self-assembled monolayers (SAMs) of APTES/AHS (3-amino propyl triethoxy silane and allyl hydroxl silane) with 0.01%, 0.1%, 1%, 10%, and 100% of concentration of APTES were tested. This leads to coatings with different density and protein distribution that can be used to test the effect of protein density distribution on thio-phosphorylation detection using our EIS/LSPR technology. In order to check the effectiveness of this new method of controlling protein immobilization by using different solution ratios of APTES/AHS, horse radish peroxidase (HRP) was used as a test protein. Initial experiments were performed by immobilization of HRP with varied concentrations of APTES and testing the levels of HRP using a TMB (3,3',5,5'-Tetramethylbenzidine) assay.

HRP was incubated on the Si_3N_4 surface at a concentration of 150 µM $^-$for 30 min. The surface was then rinsed with 1 mM Tris buffer, pH 7.4, to remove excess of HRP. 300 µl of TMB solution was dispensed on the Si_3N_4 surface and the samples were incubated at room temperature for 20 min until a distinct colour change was observed. Finally, the reaction was stopped by adding 20 µl of 0.5 M sulphuric acid and the absorbance was measurement at 450 nm using a low-volume spectrophotometer (Genova Nano, Jenway Biotech, USA).

Figure S3 shows the levels of absorbance observed for different solution rations of APTES/AHS. Higher concentration of APTES in APTES/AHS led to a higher amount of HRP immobilized on the surface of silicon nitride. However, the linearity was low (regression coefficient <0.97), especially at higher concentrations of APTES possibly due to the linear mismatch in the orientation of how SAMs are formed on the surface and the way proteins attach to the functionalized group on the SAM.

Using a similar immobilization approach, an analysis was performed on how LSPR- and EIS-based phosphorylation detection were affected by APTES concentration in the immobilization of MBP (Figure S4). Our assumption that density of MBP can be controlled in a similar way as that of HRP was validated when the EIS signal was found different at varied concentrations of APTES with optimal level only at 100% APTES; the LSPR signal at 1% 10% and 100% concentrations of APTES was found to be near-maximal. This might suggest that the amount at 1% concentrations of APTES the amount of immobilised protein is sufficient for maximal AuNP binding and saturation of the LSPR signal.

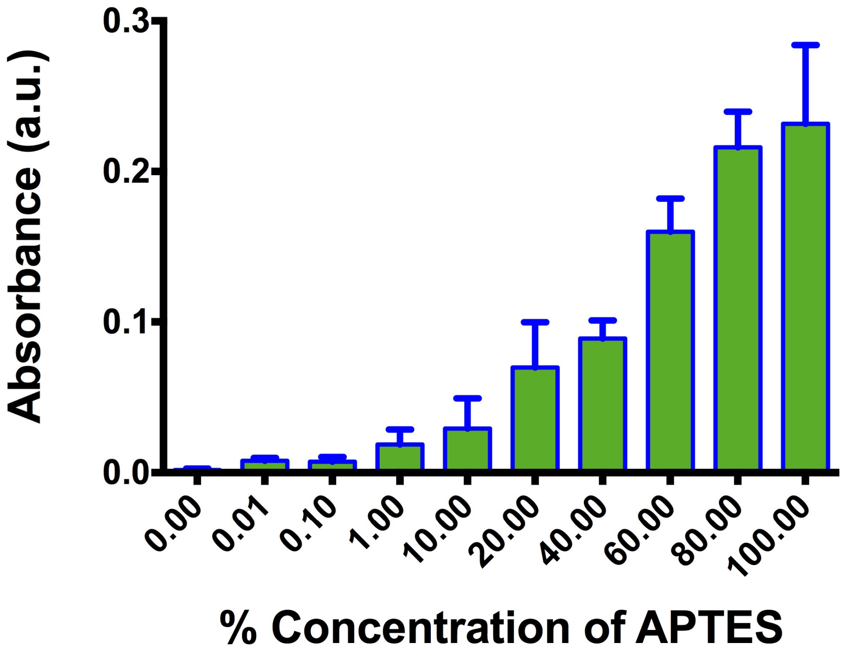

Figure S3 - TMB assay for the conformation of protein immobilization at varied concentration of APTES in APTES/AHS solution

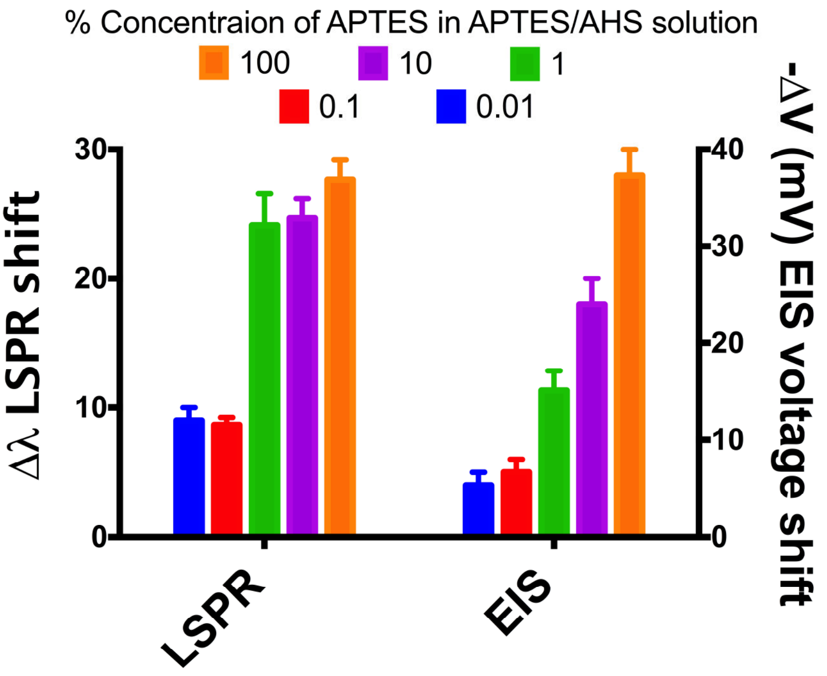

Figure S4 - EIS and LSPR response at varied concentrations of APTES in APTES/AHS solution.
